# Why focus on mental health systems?

**DOI:** 10.1186/1752-4458-1-1

**Published:** 2007-08-09

**Authors:** Harry Minas, Alex Cohen

**Affiliations:** 1 Centre for International Mental Health, School of Population Health, The University of Melbourne, Parkville, Australia; 2 Department of Social Medicine, Harvard Medical School, Boston, USA

## Abstract

The global situation for people with mental illness – in developing and developed countries – is dire. Legislative and human rights protections are frequently lacking. Mental health budgets are inadequate. There are insufficient numbers of skilled policy makers, managers and clinicians. Communities are poorly informed about mental health and illness and not well organised for purposes of advocacy. In most of the world, mental health services are inaccessible or of poor quality. Most people who would benefit from psychiatric treatment and rehabilitation do not have affordable access to such services. Leadership – at all levels – for mental health system development needs to be greatly strengthened.

While mental health research attention and funds are devoted predominantly to neuroscience and clinical research, we believe that the highest global mental health research priority is mental health systems research. There is an urgent need to focus on the development of effective, appropriate, affordable mental health services. The evidence base for such development is currently weak.

The *International Journal of Mental Health Systems *aims to stimulate greater attention to the central importance of building functioning mental health systems. Rapid publication and global reach through open access will make this journal a resource for all those who wish to contribute to such development.

## Background

**Figure 1:**
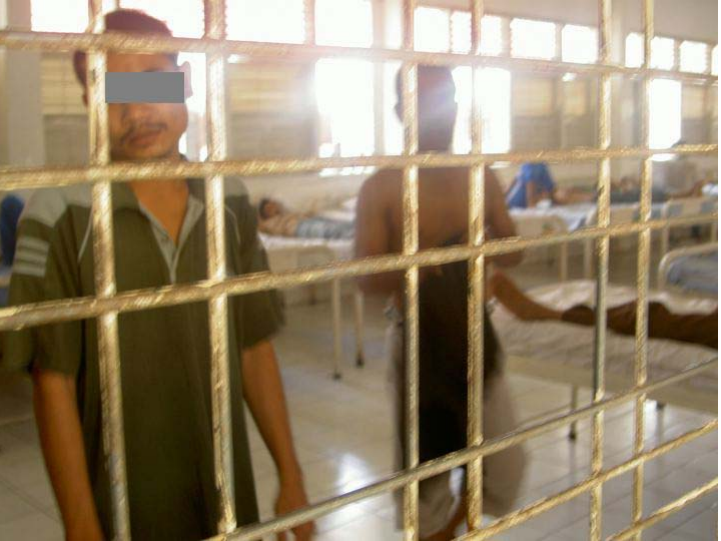
Conditions such as these are far too common in mental hospitals in many parts of the world. (Photo: Dr Harry Minas)

**Figure 2:**
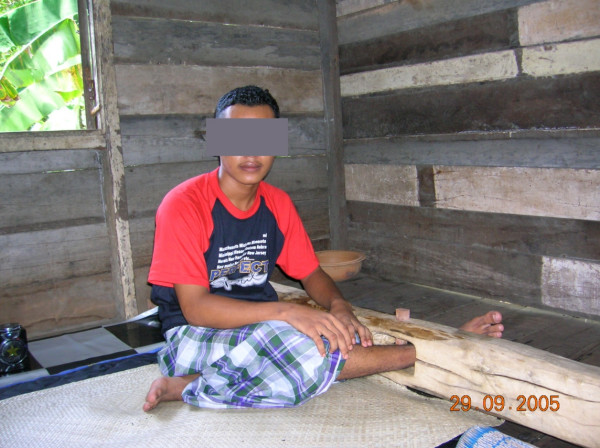
In circumstances where treatment services are lacking many people with mental illness are physically restrained in their homes by their families using a variety of means. (Photo: Dr Budi Anna Keliat)

The need for increased attention to and investment in mental health has been repeatedly highlighted, often with recommendations about what should be done [1]. Despite this, the situation for people with mental illness in low and middle-incomes countries remains dire [2]. In 2003 *Time Asia *ran a story [3] accompanied by a disturbing photoessay [4], that graphically illustrated the plight of people with mental illness is Asia. The response to the story was silence. UN Resolution 46/119 [5] was not invoked, there was no inquiry or action, and the story – and the issue of the human rights of people with mental illness, in general, – receded into oblivion. In many places hospitals lack the resources to provide effective and dignified care (Figure 1) and, in the community, families are forced by the lack of treatment and support services to restrain family members in unacceptable ways (figure 2).

Even in countries that have paid a great deal of attention to mental health system reform, and substantially increased investment in mental health services over the past decade, serious questions remain about the accessibility and quality of public mental health services [6].

In low- and middle-income countries the so-called treatment gap [7] is particularly wide, and appears to be getting wider [8], although it also exists in the richest countries with the most highly developed mental health services.

## Mental health systems research

Many people with mental illness derive no benefit from the advances that have been made in understanding of and treatments for mental illness [8] because a substantial proportion receive inadequate treatment or no treatment at all.

There is an urgent need to focus on the development of effective, appropriate, affordable and equitable mental health systems. A major impediment to the achievement of this goal is the lack of evidence for what kinds of mental health systems are appropriate and effective in varying political, social and economic contexts [9].

The research effort in mental health is committed to neuroscience and clinical research – discovering causes of and treatments for mental disorders. Of course these areas of research are vital and will continue to be vigorously pursued. However, it is well to remember that the new and better treatments that are discovered cannot be delivered in the absence of functioning mental health systems.

In coming decades, the proportion of disability attributable to mental disorders is projected to increase [10]. We believe that the highest priority in global mental health research is mental health systems research.

## Mental health system development

We use the term *mental health system *to include:

• mental health policies, plans and programs

• legislation and regulations governing mental health service organization and practice

• mental health financing and payment arrangements

• organization of service programs for detection and treatment of mental illness, including reliable supply of psychotropic medicines, and rehabilitation services

• systems for training of mental health practitioners from all relevant disciplines

• the mental health information systems that enable planning, monitoring and evaluation

• programs that are devoted to mental health promotion and illness prevention

• social arrangements that promote social participation – including work – and income support for people with mental illness

• the political, sociocultural and economic environment in which all this occurs.

Mental health system development requires attention to all of these areas and must be informed by evidence.

## Leadership

Bringing about substantial change in complex systems requires creative, sustained leadership at all levels of the system – in government, professional associations, clinical service organisations, and in communities. There is a need to foster and support the emergence of such leadership and to study the forms of leadership that are effective and constructive in different political, economic and socio-cultural settings. There is a particular need for mental health professionals to broaden their conceptual horizons beyond the purely clinical to include population health perspectives and to be active, informed and skilled participants in the pursuit of mental health system reform. Key issues in mental health system development and reform are how to improve health system performance [11] and how to promote the leadership at all levels that is required for change in complex health systems [12-14].

## International Journal of Mental Health Systems

Although important examples of mental health system development are to be found in all world regions evaluations and careful and systematic descriptions of such programs are rarely found in the scientific literature. The mental health systems literature that does exist is scattered across numerous professional journals in medicine, psychiatry, nursing, health policy, health economics, human rights, and health ethics. There is currently no single journal to which those who are engaged in mental health system development can turn for relevant research, debate and education.

*International Journal of Mental Health Systems *(IJMHS) is an open access, peer-reviewed journal that publishes articles on all aspects of mental health system development: mental health systems research, policy and other debates, case studies, and articles with educational intent that will build capacity for mental health systems research and development. The members of the Editorial Board are from 27 countries – in Africa, North and South America, East and South Asia, Europe, and Oceania – and from many disciplines.

Articles published in *IJMHS *are archived in PubMed Central, the US National Library of Medicine's full-text repository of life science literature, and also in repositories at the University of Potsdam in Germany, at INIST in France and in e-Depot, the National Library of the Netherlands' digital archive of all electronic publications.

The definition of open access published as part of the Bethesda Declaration [15] is:

"An Open Access Publication is one that meets the following two conditions:

1. The author(s) and copyright holder(s) grant(s) to all users a free, irrevocable, worldwide, perpetual right of access to, and a license to copy, use, distribute, transmit and display the work publicly and to make and distribute derivative works, in any digital medium for any responsible purpose, subject to proper attribution of authorship, as well as the right to make small numbers of printed copies for their personal use.

2. A complete version of the work and all supplemental materials, including a copy of the permission as stated above, in a suitable standard electronic format is deposited immediately upon initial publication in at least one online repository that is supported by an academic institution, scholarly society, government agency, or other well-established organization that seeks to enable open access, unrestricted distribution, interoperability, and long-term archiving (for the biomedical sciences, PubMed Central is such a repository)."

There is increasing research support for the view that publishing in open access journals has the potential to accelerate recognition and dissemination of research findings [16].

We intend that this will be the journal to which mental health system researchers, Health Ministers' advisers, policy makers, mental health consultants advising countries on mental health system development, teachers in psychiatry, nursing, psychology, social work and public health courses, clinicians involved in mental health system reform, and others will turn for the latest research and policy information on how to build equitable, accessible, efficient, high quality mental health systems.
